# Management of a Growth Hormone-Secreting Pituitary Macroadenoma Associated With Idiopathic Intracranial Hypertension and an Empty Sella

**DOI:** 10.7759/cureus.34471

**Published:** 2023-01-31

**Authors:** Hunter J King, Evan Luther, Alexis A Morell, Michael Ivan, Ricardo J Komotar

**Affiliations:** 1 Neurosurgery, Drexel University College of Medicine, Philadelphia, USA; 2 Neurological Surgery, University of Miami Miller School of Medicine, Miami, USA

**Keywords:** pituitary adenoma, csf leak, empty sella turcica, acromegaly, idiopathic intracranial hypertension (iih)

## Abstract

Idiopathic intracranial hypertension (IIH) is a ­condition associated with elevated intracranial pressure (ICP) and frequently presents with headaches, papilledema, and visual loss. Rarely, IIH has been reported in association with acromegaly. Although removal of the tumor may reverse this process, elevated ICP, especially in the setting of an otherwise empty sella, may result in a cerebrospinal fluid (CSF) leak that is exceedingly difficult to manage. We present the first case of a patient with a functional pituitary adenoma causing acromegaly associated with IIH and an otherwise empty sella and discuss our management paradigm for this rare condition.

## Introduction

Idiopathic intracranial hypertension (IIH) is a poorly understood condition that results in elevated intracranial pressure (ICP), papilledema, vision loss, and severe headaches [1]. Empty sella syndrome, defined radiographically by the replacement of the sellar contents with cerebrospinal fluid (CSF), is also frequently seen in conjunction with IIH and can result in hypopituitarism [2,3]. Although IIH has been associated with various endocrinopathies or anatomic abnormalities, it is exceedingly rare in the setting of a hormone-secreting pituitary adenoma [4]. If an inciting anatomic or hormonal disturbance is present, then therapies are usually directed at correcting this. However, treatment strategies often also focus on symptomatic relief by reducing CSF volume to decrease ICP [5]. We describe the case of a patient with IIH and acromegaly found to have a functional pituitary adenoma within an otherwise empty sella. Treatment involved ventriculoperitoneal shunting (VPS) prior to endoscopic transsphenoidal tumor resection as her elevated ICP and empty sella placed her at high risk of developing an irreparable CSF fistula.

## Case presentation

**Figure 1:**
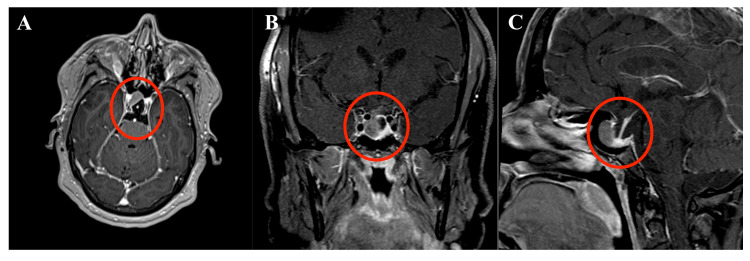
Preoperative T1-weighted MRI with gadolinium contrast illustrates a right-sided pituitary macroadenoma in an otherwise empty sella. (A) Axial, (B) coronal, and (C) sagittal plane images are displayed. MRI, magnetic resonance imaging.

A 52-year-old obese female with obstructive sleep apnea and type II diabetes mellitus presented with intermittent headaches, right-sided vision loss, and increasing finger, lip, and tongue size. On physical examination, she was found to have right-sided papilledema as well as coarse facial features concerning acromegaly. Her blood pressure was 155/73, heart rate was 80 beats per minute, respiratory rate was 19 breaths per minute, temperature was 98.4 degrees Fahrenheit, and her oxygen saturation was 100%. Her body mass index was 46.86 kg/m^2^. Magnetic resonance imaging (MRI) showed a 1.4 × 1.1 × 1.4 cm macroadenoma within the right aspect of the pituitary gland in an otherwise empty sella (Figure 1).

Her serum insulin-like growth factor 1 (IGF-1) level was 850 ng/mL (normal range: 65-216 ng/mL) confirming acromegaly. A full pituitary panel was also completed (cortisol, adrenocorticotropic hormone [ACTH], growth hormone [GH], luteinizing hormone [LSH], follicle-stimulating hormone [FSH], prolactin, thyroid-stimulating hormone [TSH], free T3, and free T4) and all were found to be within normal limits. A lumbar puncture (LP) was also performed, and the opening pressure was found to be 36 cm H_2_O confirming the concurrent diagnosis of IIH. 

**Figure 2:**
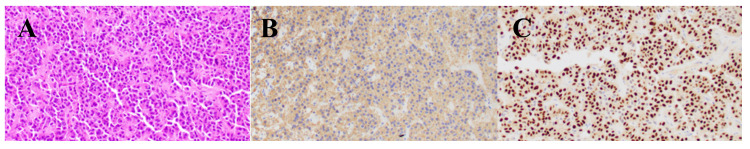
Surgical pathology shows a mixed somatotroph and gonadotroph pituitary adenoma. A. Hematoxylin & eosin staining. B. Growth hormone immunohistochemistry staining. C. PIT1 immunohistochemistry staining.

Surgical resection of the pituitary adenoma was deemed to have a significantly high risk of postoperative CSF leakage due to her elevated ICP and empty sella. Thus, VPS was performed before tumor resection. The patient then underwent endoscopic transsphenoidal pituitary tumor resection. As expected, a CSF leak occurred intraoperatively as arachnoid was immediately encountered once the sellar dura was opened. After the tumor was resected, the anterior skull base defect was repaired with an acellular dermal matrix and a nasoseptal flap. Pathology confirmed that the tumor was a mixed somatroph and gonadotroph pituitary adenoma (Figure 2).

**Figure 3:**
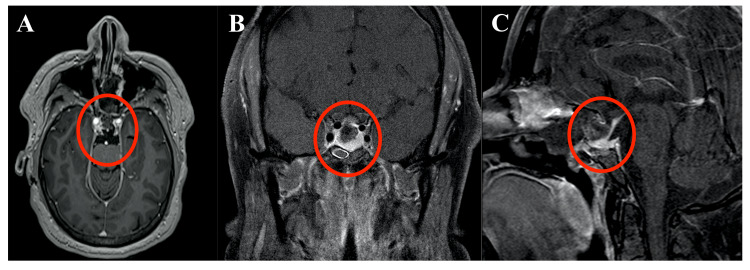
Postoperative T1-weighted MRI with gadolinium contrast demonstrates complete adenoma resection. (A) Axial, (B) coronal, and (C) sagittal plane images are displayed. MRI, magnetic resonance imaging.

Postoperative MRI is shown in Figure 3.

At one month after surgery, her IGF-1 level had decreased to 295 ng/mL and at three months she remained neurologically intact with no evidence of CSF leakage.

## Discussion

IIH, also known as pseudotumor cerebri, is a phenomenon found predominately in middle-aged, obese females. It commonly manifests with severe headaches and visual disturbances and can result in a permanent visual loss if not identified and treated appropriately [1]. Diagnostic criteria for IIH include i) signs of generalized intracranial hypertension or papilledema, ii) an opening pressure greater than 25 cmH_2_O on LP, iii) normal CSF composition, iv) and no evidence of hydrocephalus or structural lesions on imaging with v) no other identifiable cause of intracranial hypertension [6].

Another common imaging finding in IIH is an empty sella turcica [7]. Although this may result in hormonal disturbances, it is often asymptomatic and thought to occur due to herniation of the arachnoid into the sella due to chronically elevated ICP [2,3,8-12]. Although the endocrinopathies associated with an empty sella should be corrected if present, treatment of the underlying IIH may ultimately help reverse them long-term. Consequently, the treatment of IIH must consider these multifactorial components in order to optimize patient outcomes. 

Treatment options for IIH often include decreasing CSF production medically or CSF diversion either through serial LPs or surgically through shunting. Studies have demonstrated that CSF shunting can significantly improve papilledema, headache, and vision changes and should be considered in patients who experience spontaneous CSF rhinorrhea or major visual alterations due to IIH [8,13,14].

Intraoperative CSF leakage is relatively common during transsphenoidal pituitary surgery and can be incredibly difficult to repair in the setting of increased ICP [15,16]. Postoperatively, CSF fistulas are also much more common if the elevated ICPs are left uncorrected [13,17,18,19]. Despite these findings, reports of hormone-secreting pituitary adenomas and concurrent IIH remain limited and no consensus on the management of these simultaneous conditions exists.

Although IIH has been described in patients receiving recombinant GH, only one case of IIH secondary to a somatotrophic pituitary adenoma has ever been reported [4,20]. In the former cases, the IIH resolved after cessation of GH supplementation [20]. For the latter, acetazolamide was used preoperatively to medically manage the elevated ICP and five months after tumor resection the IIH resolved [4]. These findings suggest that excess circulating GH may have some effect on CSF production or reabsorption, and that removal of the inciting etiology may result in the reversal of the IIH syndrome. However, in the previous case of acromegaly associated with IIH, the sella was full, making the risk of CSF leak much lower during tumor resection [4]. As previously mentioned, our patient had an adenoma within an otherwise empty sella, placing them at exceedingly high risk of a CSF leak. Given that the IIH symptoms may not resolve for several months after tumor removal, it was determined that she should undergo CSF diversion prior to tumor removal to reduce the risk of developing an unrepairable postoperative CSF fistula. 

Our case demonstrates that caution must be taken when considering treatment for patients with concomitant IIH and acromegaly. VPS was the safest approach prior to tumor resection in a patient with an otherwise empty sella to prevent an irreparable CSF leak and reduce the potential for developing postoperative meningitis.

## Conclusions

The diagnosis of IIH associated with acromegaly is a rare phenomenon with only one other reported case in the literature. The patient in our case presented with papilledema, signs and symptoms of acromegaly, and a partially empty sella. To avoid an irreparable CSF leak in the setting of elevated ICPs, VPS was performed prior to transsphenoidal tumor resection. The presence of an empty sella in the setting of IIH and a pituitary adenoma should prompt the surgeon to contemplate CSF diversion prior to surgical resection of the tumor thereby reducing the risk of CSF leakage and subsequent meningitis.
